# Tunisian *Silybum* Species: Important Sources of Polyphenols, Organic Acids, Minerals, and Proteins across Various Plant Organs

**DOI:** 10.3390/plants13070989

**Published:** 2024-03-29

**Authors:** Samah Maaloul, Maher Mahmoudi, Hédi Mighri, Imen Ghzaiel, Talel Bouhamda, Fayçal Boughalleb, Adil El Midaoui, Anne Vejux, Gérard Lizard, Raoudha Abdellaoui

**Affiliations:** 1 Laboratory of Rangeland Ecosystems and Valorization of Spontaneous Plants and Associated Microorganisms (LR16IRA03), Arid Regions Institute, University of Gabes, Medenine 4119, Tunisia; samah.maaloul@gmail.com (S.M.); mahmoudi.maher@fst.utm.tn (M.M.); mighri.hedi@yahoo.com (H.M.); boughalleb.fay@gmail.com (F.B.); 2 Laboratory of Plant, Soil and Environment Interactions (LR21ES01), Faculty of Sciences of Tunis, University of Tunis El-Manar, Tunis 2092, Tunisia; 3 Team ‘Biochemistry of the Peroxisome, Inflammation and Lipid Metabolism’ (EA7270), University of Bourgogne, 21000 Dijon, France; imenghzaiel93@gmail.com (I.G.); anne.vejux@u-bourgogne.fr (A.V.); 4 Advanced Analysis Platform, Arid Regions Institute, University of Gabes, Medenine 4119, Tunisia; talel04091980@gmail.com; 5 Department of Pharmacology and Physiology, Faculty of Medicine, University of Montreal, Montreal, QC H3C 3J7, Canada; adil_elmidaoui@yahoo.ca; 6 Department of Biology, Faculty of Sciences and Techniques of Errachidia, Moulay Ismail University of Meknes, Meknes 50050, Morocco

**Keywords:** *Silybum marianum*, *Silybum eburneum*, minerals, polyphenols, organic acids, storage proteins, antioxidants

## Abstract

*Silybum marianum* and *Silybum eburneum* are wild edible Mediterranean plants used in the human diet. This study presents the initial findings on the phytochemical characterization of Tunisian *S. marianum* and *S. eburneum* organs. It examined their mineral, sugar, organic acid, polyphenolic, and seed storage protein contents, as well as their antioxidant potential. In *S. marianum*, stems had high sodium and potassium contents, while the immature and mature seeds were rich in calcium and magnesium. However, *S. eburneum* had high potassium levels in stems and high sodium and calcium levels in the flowers. *S. marianum* showed substantial fructose variation among its organs. Conversely, *S. eburneum* exhibited significant heterogeneity in glucose, sucrose, and maltose levels across its organs, with maltose exclusively detected in the immature seeds. A notable organ-dependent distribution of organic acids was observed among the two species. Higher levels of phenolic contents were detected in both mature and immature seeds in both species compared to the other plant parts. The seeds possessed higher antioxidant activities than other plant organs. In both *S. marianum* and *S. eburneum* seeds, albumins and globulins were the predominant protein fractions. This study brings evidence supporting the important potential of *Silybum* organs as sources of nutrients with antioxidant properties for producing functional food.

## 1. Introduction

Food presents a significant challenge for sustainable development, particularly in the eradication of world hunger and redirection of agricultural practices toward the goals of providing access to food for all, ensuring quality, and respecting the environment [1]. To achieve this, it is crucial to combine technological innovation with social and cultural innovation to produce food that meets the nutritional, personal, and social needs of all communities. Functional food is considered one of the most intriguing areas of research and innovation in the food industry [2,3]. Functional foods are a new type of food that is formulated to contain, in addition to nutrients, natural substances or microorganisms that have a beneficial effect on health [4]. Functional foods can be classified into four categories: fortified products, enriched products, altered products, and enhanced commodities. Fortified products are foods that have additional nutrients added to them, such as fruit juices fortified with vitamin C or E. Enriched products are foods that have new nutrients or components added that are not usually found, such as probiotics or prebiotics. Modified food products are those where a harmful component has been removed, reduced, or replaced with a beneficial one to reduce an existing health risk. Additionally, enriched products are those in which one of the components has been naturally increased, such as eggs with higher omega-3 contents [2,3]. The major compounds added to functional foods, including carbohydrates, proteins, fibers, bioactive compounds, and vitamins, are often extracted from plants [5]. In the Mediterranean basin, especially in the arid zones of Tunisia, there are several endemic plants that contain functional compounds. However, these plants have not yet been well developed. One such plant is the *Silybum* genus.

The *Silybum* genus is classified within the tribe *Asterales* of the *Asteraceae* family and is native to the Middle East, the Arabian Peninsula, the Indian subcontinent, North Africa, and certain regions of Europe. It has been introduced to various regions, including Japan, sub-Saharan Africa, North and South America, parts of Europe, Australia, and New Zealand [6]. The genus *Silybum* comprises two closely related species: *S. marianum* (SM) is characterized by its variegated foliar morphology and *S. eburneum* (SE) exhibits a uniform green leaf [7]. Initially considered distinct species, genetic studies have since revealed that these two variants are closely related and likely represent different forms or varieties within the same species [8,9]. *S. eburneum* is a non-grass herb, a wild plant characterized by its glabrous, spine-scented nature. It typically thrives as an annual or biennial species, predominantly found in marginal land, grassy banks, and river flats. This erect plant can reach heights ranging from 15 to 300 cm. It produces solitary, light purple (occasionally white) flowers, often in profusion, from the months of June through September. The leaves are uniformly green, simple, and arranged alternately along the stem. They are lanceolate in shape, featuring denticulate margins and petiolate attachments. The seeds are notably hard and come equipped with a white, silky pappus for dispersal [9]. For *S. marianum,* leaves are arranged alternately and are notable for their significant size and glabrous texture, featuring spiny margins. These leaves typically measure between 50 and 60 cm in length and from 20 to 30 cm in width [10]. The plant’s stem exhibits a height range of 40 to 200 cm, displaying a smooth or slightly downy texture. It maintains an erect posture and undergoes branching primarily in the upper section of the plant [11]. Each stem culminates in a flower head, approximately 5 cm in diameter, displaying a distinctive red-purple hue. The inflorescences are encircled by protective spiny bracts [12]. The seeds are achenes measuring 5–8 mm in length, featuring an elongated white pappus and exhibiting a color spectrum spanning from black to brown [13]. The weight of 1000 seeds ranges from 28 to 30 g. Each flower head yields approximately 190 seeds, resulting in an average seed production of 6350 seeds per individual plant [14]. Furthermore, it is noteworthy that seeds can maintain viability in the soil for as long as nine years [15].

For centuries, various local rural communities in the Mediterranean basin have incorporated these plants into their diets. In Italy, the peeled heads and stems are consumed raw [16]. In Spain, *S. marianum* has a historical tradition of culinary use as a salad vegetable or as a boiled and fried dish [17]; Arabs have a tradition of consuming the young fleshy stems, seeds, and sprouts [18]. In Tunisia, these plants are listed among the widely consumed plants, and the edible parts consist of the capitulum (flower head) and seeds. These two components are commonly consumed either in their raw form or are utilized as essential ingredients in traditional breakfast cereals: “Bsissa” [19]. This plant is listed among the most highly valued wild edible plants, recognized for its potential as a food ingredient with significant economic value [20]. The mid-ribs of basal leaves are widely consumed, either raw or cooked, in several Mediterranean countries [21]. The basal leaves are commonly stewed, and rarely used in their raw form as ingredients in salads [22].

The milk thistle seeds have been extensively employed in medicinal contexts for an impressive span of over two millennia, principally acclaimed for their therapeutic effectiveness in addressing liver-related maladies [23]. Ref. [24] noted that milk thistle received much attention as an innovative and versatile crop for agriculture in challenging environments. Additionally, the fruits of this plant are industrially utilized in the production of silymarin [25]. It is proven that the seeds of *S. marianum* contain a high amount of oil (between 11.69 and 29.68%), proteins (20.35 to 25.25%), carbohydrates (38.16%), and fiber (27.24 to 29.95%) and possess numerous beneficial components such as essential amino acids, minerals, and phytochemicals with potential biological activities such as antioxidant and antimicrobial effects [25,26,27].

The primary aim of this study was to perform a comparative analysis of the phytochemicals extracted from different plant organs, including the leaves, stems, flowers, immature seeds, and mature seeds of *S. marianum* and *S. eburneum*, which naturally grow in Tunisia. This is the first report on *S. eburneum* compared to *S. marianum*. Profiles of minerals, soluble sugars, organic acids, and polyphenols (total phenol and flavonoid contents and phenolic acids) were investigated. Additionally, the secondary aim of this study was to determine the antioxidant activity of the extracts. This was assessed through three in vitro methods: total antioxidant activity [28], DPPH (2,2-diphenyl 1-picrylhydrazyle) free radical scavenging activity [29], and reducing power assay [30]. In addition, the protein storage of the mature and immature seeds of the two species was also analyzed.

## 2. Results

### 2.1. Mineral Composition in Silybum marianum and Silybum eburneum Organs

The mineral analysis conducted on two plant species, *S. marianum* and *S. eburneum*, revealed intriguing insights into the distribution of essential elements across various plant parts. Table 1 shows that calcium is the most abundant mineral element in both species, with levels ranging from 100.5 to 326.9 mg/100 g dry weight (DW) in *S. marianum* stems and immature seeds, respectively, and from 106.9 to 230.2 in *S. eburneum* mature seeds and flowers, respectively. There was no significant difference between the two species in calcium levels (*p* = 0.912). However, there was a highly significant difference observed between organs, regardless of plant type, and also between the same organs of the two plants.

In *S. marianum*, sodium and potassium contents were highest in the stem (19.8 and 44.2 mg/100 g DW, respectively) and leaves (18.7 and 39.6 mg/100 g DW, respectively), while calcium and copper were most abundant in the immature seeds (326.9 and 0.1 mg/100 g DW, respectively). Magnesium, however, exhibited a relatively lower but varied presence in different parts, with the highest concentration observed in mature seeds and leaves (5.1 mg/100 g DW for both). Furthermore, the leaves were significantly richer in iron and manganese. In *S. eburneum*, a non-significant difference was observed between the organs in sodium levels (*p* = 0.153), which ranged from 41.4 to 64.9 mg/100 g DW. In addition, stems and flowers were significantly richer in potassium levels. Flowers and leaves contained the highest calcium contents (230.2 and 224.6 mg/100 g DW, respectively). Similarly, magnesium contents displayed variations across plant parts, with the highest levels observed in the leaves and stems. There was a higher concentration of copper in flowers and both mature and immature seeds, while no significant differences were observed between organs in terms of iron and manganese levels (*p* = 0.232 and *p* = 0.130, respectively).

### 2.2. Free Sugar Composition in Silybum marianum and Silybum eburneum Organs

Table 2 summarizes the carbohydrate composition of *S. marianum* and *S. eburneum* across various organ types. The investigation elucidated critical components within these plants, encompassing fructose, glucose, sucrose, and maltose. This profiling revealed noteworthy differentials in the chemical constituents of the examined organs, with highly significant differences between species. *S. marianum* exhibits high significant variation in fructose and sucrose contents across its organs, with values ranging from 0.01 mg/g DW in mature seeds to 3.5 mg/g DW in stems and from 0.1 mg/g DW in immature seeds to 0.6 mg/g DW in mature seeds, respectively. No significant difference was observed in glucose contents (*p* = 0.099). In contrast, *S. eburneum* demonstrated considerable heterogeneity in fructose, glucose, and maltose levels in its corresponding organs. For instance, *S. eburneum*’s leaves, stems, and flowers displayed the highest fructose contents, which ranged from 4.8 mg/g DW (in flowers) to 5.5 mg/g DW (in stems). The highest levels of glucose were detected in leaves (3.8 mg/g DW) and mature seeds (3.6 mg/g DW). No significant difference was detected in sucrose levels (*p* = 0.099). Maltose was only detected in the immature seeds of *S. e burneum.* Significant differences emerged clearly for fructose and maltose. However, for glucose and sucrose, significance was noted in species effect (*p* = 0.034 and *p* < 0.0001, respectively), but the main effects of organs (*p* = 0.059 and *p* = 0.176, respectively) and species–organ interaction (*p* = 0.059 and *p* = 0.132, respectively) were not statistically significant (Table 3).

### 2.3. Organic Acid Contents in Silybum marianum and Silybum eburneum Organs

The organic acid contents in different organs of *S. marianum* and *S*. *eburneum* are summarized in Table 4. While oxalic acid and quinic acid were not detected in parts of *S. marianum*, their contents varied significantly in *S. eburneum*. The leaves contained a higher level of oxalic acid (0.03 mg/g DW), while the flowers and stems contained a higher level of quinic acid (1.1 mg/g DW for both). Among *S. marianum* organs, citric acid was only detected in the leaves at a concentration of 6.5 mg/g DW. In contrast, its content varied significantly (*p* = 0.023) from 0.1 to 0.3 mg/d DW in the investigated parts of *S*. *eburneum.* Malic acid levels varied in response to plant part and species, with amounts ranging from 0.3 (mature seeds) to 15.0 mg/g DW (leaves) for *S. marianum* and from 0.1 to 0.6 mg/g DW in the mature seeds and flowers of *S*. *eburneum*, respectively. Succinic acid showed variability across plant parts. The concentrations in leaves were highest in both species: 1.0 mg/g DW for *S. marianum* and 1.2 mg/g DW for *S. eburneum*. However, succinic acid was not detected in the immature seeds of either species nor in the mature seeds of the first species or the stems of the latter species. Lactic acid was present only in the seeds of *S. eburneum*, with a concentration of 0.2 mg/g DW in both mature and immature seeds. In *S. marianum*, this acid was found in all parts, with concentrations varying between 0.2 and 3.7 mg/g DW in the stems and immature seeds, respectively. Formic acid exhibited varying concentrations across plant parts, with values ranging from 0.04 to 0.50 mg/g DW in *S. marianum* and from 0.2 to 0.4 mg/g DW in *S. eburneum*. Higher amounts of acetic acid were detected in the leaves of both plants, measuring 37.3 and 38.8 mg/g DW for *S. marianum* and *S. eburneum*, respectively. Propionic acid was detectable in all plant parts of *S. marianum*, with values ranging from 0.2 to 0.5 mg/g DW. This acid was not detected in *S. eburneum* stems and mature seeds.

### 2.4. Storage Protein Content in the Mature and Immature Seeds of S. marianum and S. eburneum

**Figure 1:**
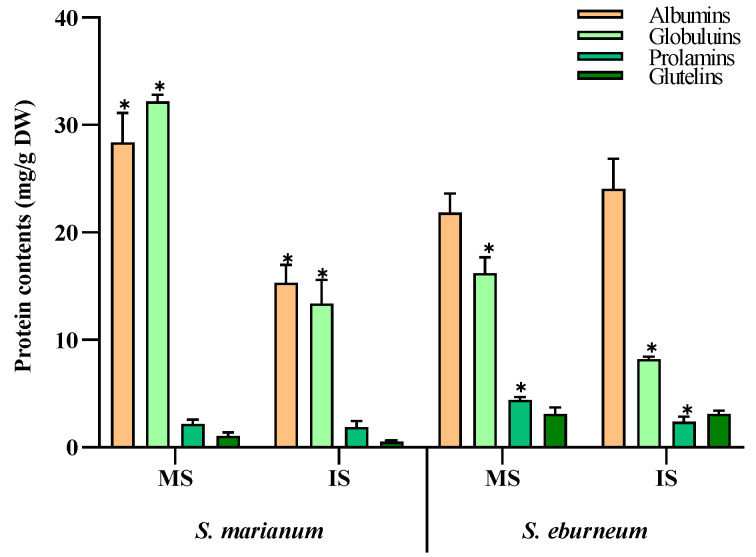
Storage protein contents (mg/g DW) in mature and immature seeds from *Silybum marianum* and *Silybum eburneum*. Values are expressed as mean ± SD (n = 3). *: significant difference between mature seeds and immature seeds in the same compounds per species (*p* < 0.05; S.N.K test). MS: mature seeds; IS: immature seeds.

In both *S. marianum* and *S. eburneum* seeds, albumins and globulins were the predominant protein fractions in both mature and immature seeds (Figure 1). In mature *S. marianum* seeds, albumins accounted for 28.4 mg/g DW and globulins for about 32.2 mg/g DW. However, in mature *S. eburneum* seeds, albumins comprised about 21.9 mg/g DW and globulins about 16.2 mg/g DW. Additionally, there was significant variation in the levels of prolamins and glutelins between the two plants, especially in mature seeds. Mature *S. eburneum* seeds exhibited the highest levels of prolamins and glutelin, measuring 4.4 and 3.1 mg/g DW, respectively.

### 2.5. Phenolic Profiles and Antioxidant Activities of S. marianum and S. eburneum Organs

#### 2.5.1. Spectroscopy Analysis

The results obtained by the Folin–Ciocalteu and chloride ammonium assays revealed that total phenol and flavonoid contents varied considerably across the different plant parts of *S. marianum* and *S. eburneum* (Table 5). For both species, the highest phenol amounts were observed in the mature seeds, with *S. marianum* showing a substantially higher value (161.4 mg garlic acid equivalent (GAE)/g dry extract (DE)) compared to *S. eburneum* (57.9 mg GAE/g DE). In contrast, *S. eburneum* exhibited significantly higher phenol contents in the flowers and stems (24.6 and 4.7 mg GAE/g DE) compared to *S. marianum* (5.9 and 0.7 mg GAE/g DE), respectively. The total flavonoid contents varied significantly between both species. Our findings indicate that *S. marianum* displayed a substantially higher content of flavonoids in their organs compared to *S. eburneum*, with the contents ranging from 2.9 to 41.9 mg quercetin equivalence (QRE)/g DE for the stems and mature seeds, respectively. These contents were in the range of 3.6–17.5 mg QRE/g DE in *S. eburneum* leaves and mature seeds, respectively. Statistical analysis revealed strong statistical evidence for the significant effects of species, organs, and their interaction on the measured phenol and flavonoid contents (Table 3).

#### 2.5.2. Liquid Chromatography Coupled with Mass Spectroscopy Analysis of Phenolic Acid Compounds

The different organs of *S. marianum* and *S*. *eburneum*, including its leaves, stems, flowers, mature seeds, and immature seeds, were further submitted to liquid chromatography coupled with mass spectroscopy and electrospray ionization system (LC-ESI/MS) analysis (Table 6) for a qualitative and quantitative investigation of the phenolic acid compounds. A total of 22 phenolics were tentatively identified including 16 phenolic acids and 16 flavonoids. For *S. marianum*, among the detected phenolic acids, 3,4-di-O-caffeoylquinic acid was found in substantial quantities in stems (3402.4 µg/g DE), leaves (11,405.2 µg/g DE), and flowers (20,538 µg/g DE), and the highest concentration was observed in the flowers. However, this compound was not detected in either immature or mature seeds. Protocatechuic acid was significantly higher in the flowers (4133.5 µg/g DE) and leaves (326.4 µg/g DE), while 4,5-di-O-caffeoylquinic acid was higher in the leaves (2269.1 µg/g DE) and stems (503.2 µg/g DE). Quinic acid was most prevalent in the mature seeds (10404.5 µg/g DE) and immature seeds (6389.3 µg/g DE) and lower in the flowers (357.9 µg/g DE), stems (104 µg/g DE), and leaves (72.1 µg/g DE). Additionally, o-coumaric acid was prevalent in the flowers and stems and not detected in the other plant parts. Trans cinnamic acid was present only in mature seeds (7319.9 µg/g DE) and stems (482.1 µg/g DE). Apigenin was not detected in the mature seeds. Quercetin was present at higher levels, and it was found in the range of 3.4 to 1814 µg/g DE for leaves and immature seeds, followed by naringenin, which varied from 24.01 µg/g DE to 1694.4 µg/g DE for stems and immature seeds, respectively. Quercetrin and quercetin-3-o-galactoside were prevalent in the flowers and immature seeds. For *S*. *eburneum* parts, the levels of identified polyphenolic compounds were lower than those detected in the *S. marianum* organs. In the leaves, chlorogenic acid was the main compound (82.4 µg/g DE), followed by apegenin-7-o-glucoside (63.6 µg/g DE). In the stems, the contents of syringic acid (168.1 µg/g DE) and apegenin-7-o-glucoside (139.9 µg/g DE) were the highest. In the flowers, 13 compounds were found, mostly kaempferol (1281.3 µg/g DE), apigenin (877.5 µg/g DE), naringenin (160.1 µg/g DE), syringic acid (110.02 µg/g DE), protocatechuic acid (56.7 µg/g DE), quinic acid (34.2 µg/g DE), and chlorogenic acid (33.9 µg/g DE).

#### 2.5.3. The Antioxidant Activities in the Different Parts of *S. marianum* and *S. eburneum*

The antioxidant potential in different parts of the *S. marianum* and *S*. *eburneum* plants was determined, with a focus on three key components: total antioxidant activity (TAA), free radical DPPH scavenging activity, and reducing power assay (Table 5). In *S. marianum* extracts, both mature and immature seeds possessed higher TAA (24.7 and 31.2 mg GAE/g DW). Also, the flowers showed higher TAA (28.6 mg GAE/g DW) compared to the leaves (19.9 mg GAE/g DW) and stems (8.1 mg GAE/g DW). In *S*. *eburneum*, the flower part had the highest TAA (22.6 mg GAE/g DW), which was 5.73-fold higher than that of the stems, 4.91-fold higher than that of immature seeds, and about 3.8-fold higher than that of both leaves and mature seeds. Furthermore, *S. marianum* immature seeds and *S*. *eburneum* flowers exhibited the highest DPPH radical scavenging activities, measuring 4.4 and 2.5 mg Trolox equivalent (TRE)/g DW, respectively. At the same time, the highest reducing power assays were observed in the mature seeds of both species (44.9 mg TRE/g DW for *S. marianum* and 16.0 mg TRE/g DW for *S. eburneum*). Statistical analysis showed significant differences in the effects of species, organs, and their interaction on total antioxidant capacity, DPPH, and FRAP assays (Table 3).

### 2.6. Statistical Effects of Species, Organs, and Species–Organs Interaction 

To further investigate the effects of species, organs, and their interaction, Student’s *t* test was conducted on the phytochemical profiles, including minerals, sugars, organic acids, phenolic acids, and total phenol and flavonoid contents, and antioxidant activities of *S. marianum* and *S. eburneum*. Table 3 illustrates a highly significant difference between the two species, as well as between the different organs whatever the species, and between the same organs of the two species for most of the analyzed tests.

There was a significant difference in glucose and sucrose contents between species, but non-significant effects of organs and species–organs interaction. Regarding organic acids, only acetic acid showed no significant difference between the two species. In terms of minerals analyses, only calcium content showed no species effect, while sodium exhibited no significant difference in the interaction effect. On the other hand, phenolic acid profiles did not show any significant effects of species, organs, or the interaction between species and organs in the majority of compounds. But, 15 phenolic acids showed a highly significant difference between species, organs, and their interaction.

## 3. Discussion

The genus *Silybum* comprises only two species: *S. marianum* and *S. eburneum*, which can be considered wild edible plants. *S. marianum* is renowned for its diverse biological activities and pharmacological and medicinal properties. While *S. marianum* has been extensively characterized worldwide in terms of phytochemical and biological activities, our knowledge regarding *S. eburneum* is quite scarce in comparison. Recent research has solely focused on the seeds (achenes) of milk thistle, which are rich in active substances, particularly silymarin and silybin. This work presents the first phytochemical report of different organs of Tunisian *S. marianum* and *S. eburneum*. This study primarily focuses on the profiles of mineral elements, sugars, organic acids, and phenolic compounds (phenolic acids and total phenol and flavonoid contents) as well as the antioxidant activities (total antioxidant activity, DPPH, and FRAP). Additionally, the protein fractions of mature and immature seeds were also investigated.

The mineral element profiles reveal a significant effect of species, organs, and their interaction. Calcium was the major compound detected in both species’ organs. Additionally, *S. eburneum*’s different organs had higher sodium, potassium, and magnesium contents. In contrast, copper, iron, and manganese were detected at low levels in both species. These findings shed light on the mineral composition of these plants and have implications for agricultural and nutritional studies. In their study, García-Herrera et al. observed a significant variation in the mineral concentrations found in the leaves of *S. marianum*: Na spanned from 24.7 to 128.0 mg/100 g, K from 432 to 1300 mg/100 g, Ca from 42 to 171 mg/100 g, Mg from 10.3 to 22.6 mg/100 g, Cu from 0.01 to 0.17 mg/100 g, Fe from 0.47 to 0.55 mg/100 g, Mn from 0.03 to 0.21 mg/100 g, and Zn from 0.21 to 0.35 mg/100 g [21]. Additionally, Ghafor et al. suggested that the stems of *S. marianum* also could be a potential source of minerals, with their highest concentrations of Si and Al [31].

On the other hand, a highly significant difference between the two species was observed in the sugar profiles. *S. marianum*’s organs were rich in glucose (ranging from 0.5 to 17.7 mg/g DW), while fructose and sucrose were dominant in *S. eburneum*’ organs (ranging from 0.3 to 5.5 mg/g DW and from 1.1 to 3.3 mg/g DW, respectively). Maltose was only detected in *S. eburneum* immature seeds. These results are higher in comparison with Denev et al., where the fructose, glucose, and sucrose contents in the defatted *S. marianum* seeds were 0.1139, 0.168, and 1.6433 mg/g, respectively [27]. The free sugars in the aerial parts of *S. marianum* have been widely discussed. Tian et al. and Zhauynbaeva et al. reported the presence of glucose, galactose, mannose, rhamnose, xylose, and arabinose [32,33]. Additionally, mannitol, sucrose, fructose, raffinose, arabinose, and galactose were detected in the stems of the plant [31]. The organ-specific disparities in sugar contents observed in our study are in alignment with the findings of Eldalawy et al., who reported varying concentrations of fructose, glucose, and myo-inositol in different parts of the flowers, leaves, and seeds [34].

In the plants, organic acids are involved in several fundamental pathways, including as intermediate or end products in catabolic and metabolic pathways [35]. Some of them, like malic, citric, and oxalic acids, could be related to processes operating within the rhizosphere, such as nutrient acquisition, metal detoxification, the mitigation of anaerobic stress in root systems, and mineral weathering [36]. In our study, oxalic, quinic, and citric acids were detected only in the organs of *S. eburneum*. In addition, a significant difference between species was observed in malic, succinic, lactic, formic, and propionic acids. The quinic, malic, shikimic, citric, and fumaric acids were previously described in the aerial parts of *S. marianum*, with a total value of 53 mg/g DW [37]. Organic acids are classified as weak acids on a chemical level. They have been widely used in food preservation for centuries. Recently, organic acids, such as formic, butyric, propionic, acetic, citric, malic, and lactic acids, have been reported for their potential antibacterial and immune potentiating properties [38]. In their study, Sánchez-Mata et al. observed that among several edible plants, *S. marianum* exhibited the highest values for total organic acids, with the highest contents of oxalic acid (662.03–464.50 mg/100 g) and fumaric acid (2.96–26.29 mg/100 g) [39]. Malic and citric acids were only detected in one population, with levels of 1.69 and 1.49 mg/100 g, respectively. Our findings align with those of Pereira et al., who suggested that *Silybum* species could be incorporated into food formulations as acidulants, owing to the abundant presence of these organic acids in various parts of the plants [37].

For the protein fractions, the results indicate that albumins and globulins are the predominant protein fractions in the seeds of *S. marianum* and *S. eburneum*. Furthermore, there was significant variation in the levels of prolamins and glutelins between the two plants’ seeds. In the study of Li et al., it was noted that albumin was the predominant fraction, followed by globulin, with smaller amounts of glutelins and prolamins [40]. The seeds of *S. marianum* are known for their accumulation of silymarin in the pericarp and seed coat. This compound is well known for its detoxifying effect and ability to stabilize liver functions [28,29]. As a result, the plant has been widely cultivated for pharmaceutical purposes in several countries. The seeds contain a high amount of total protein, measuring 16.5% [41] and 19.1 g/100 g [18]. According to Zhu et al., the proteins found in *S. marianum* seeds contain high levels of glutamic acid and essential amino acids, ranging from 32.33 to 38.24 g per 100 g of protein [42]. These levels meet the FAO/WHO requirements for infants aged 2 to 5 years. No allergic reactions to milk thistle proteins have been reported. Shahat et al. showed that incorporating defatted milk thistle seed flour at a 3% level in wheat bread has the potential to improve bread characteristics [43]. That is why defatted milk thistle seeds in flour form could be effectively used in functional food production.

In addition to the profiles of mineral elements, sugars, organic acids, and seed protein fractions, phenolic profiles were investigated. In this study, the total phenol and flavonoid contents varied significantly between species, organs, and their interaction. The mature seeds of both species had the highest amounts of phenols, with 161.4 mg GAE/g DE in *S. marianum* and 57.9 mg GAE/g DE in *S. eburneum*. According to Guemari et al., for total phenol contents in the Algerian *S. marianum*, the highest value was recorded in seed extracts (127.39 mg GAE/g DW), followed by the flowers (42.22 mg GAE/g DW), leaves (22.25 mg GAE g DW), and twigs (9.05 mg GAE/g DW) [44]. Also, the flower and seed parts possessed higher amounts of flavonoids (34.06 and 19.41 mg EQ/g DW) compared to the other plant parts. In the seeds of *S. marainum*, the polyphenol and flavonoid contents were found to be 29 mg GAE/g DW and 3.39 mg EC/g DW, respectively [45]. Ali et al. found that polyphenol and flavonoid contents in *S. marianum* seeds were 245.183 mg GAE/g DW and 88.151 mg quercetin/g DW, respectively [46]. Furthermore, Lucini et al. revealed that the milk thistle genotypes contained polyphenols within the range of 206–360 mg GAE per 100 g achenes [47]. Moreover, Aziz et al. noted that the total polyphenols in the seeds varied from 24.17 to 35.07 mg GAE/g, while the flavonoids varied from 16.01 to 29.09 mg QRE/g [48]. It is well known that the extraction of phytochemicals from *S. marianum* is highly influenced by both the type of solvent and the extraction method, as evidenced by ref. [49].

Furthermore, the leaves, stems, flowers, mature seeds, and immature seeds of *S. marianum* and *S. eberneum* plants were analyzed using LC-ESI/MS to investigate the phenolic acid profiles. A total of twenty-two phenolics were tentatively identified, with no significant difference found between species, organs, or their interaction for the majority of the compounds detected. In their analysis of phenolics in 15 genotypes of *S. marianum*, Lucini et al. found varying concentrations of phenolic acids in the extracts, with notable amounts of chlorogenic acid (148–361.6 mg/kg), caffeic acid (2.2–33.6 mg/kg), and ferulic acid (9.7–26.5 mg/kg) [47]. Apigenin (2–11.9 mg/kg) and luteolin (3.5–79.7 mg/kg) were identified as the most abundant flavonoids, while luteolin 7-O-glucoside and quercetin were absent in all genotypes studied. In addition, Sadowska et al. identified some phenolic compounds in *S. marianum* seeds, like isosilybin A (21.9%), silybin B (17.67%), isosilybin B (12.8%), silybin A (12.2%), silychristin (7.9%), and silydianin (7.5%) [41]. Also, the authors reported higher contents of silybin that varied from 3086 to 9499 mg/kg.

In addition, this study also aimed to investigate the antioxidant activities of different organs of *S. marianum* and *S. eburneum* in terms of their phenolic compound contents, including total phenol and flavonoid contents as well as phenolic acids. Three tests were conducted: total antioxidant activity, DPPH, and FRAP. The present study also shows a significant effect of both species and organs on antioxidant activities. The data obtained in the present study are supported by several previous studies that have shown that the seeds exhibit higher antioxidant activity. For instance, it was reported by Ahmad et al. that the seeds of the Pakistan milk thistle possessed the greatest antioxidant potential compared to the stems, leaves, and roots [50]. Also, with the Algerian *S. marianum*, the seeds were found to possess the highest total antioxidant capacity, while the leaf extract exhibited the lowest activity. Additionally, the seeds demonstrated the most significant DPPH radical activity, with twigs showing the lowest potential [44]. As evidenced by Aziz et al., the seeds of *S. marianum* possessed high DPPH radical scavenging activities that varied from 18.9 to 25.01% and potential FRAP that was in the range of 9.73–17.69 mg ascorbic acid equivalents [48]. Interestingly, these data agree with the important antioxidant activities of *S. marianum* seed oils from different areas of Tunisia that have been previously described [51,52].

## 4. Materials and Methods

### 4.1. Plant Collection

**Figure 2:**
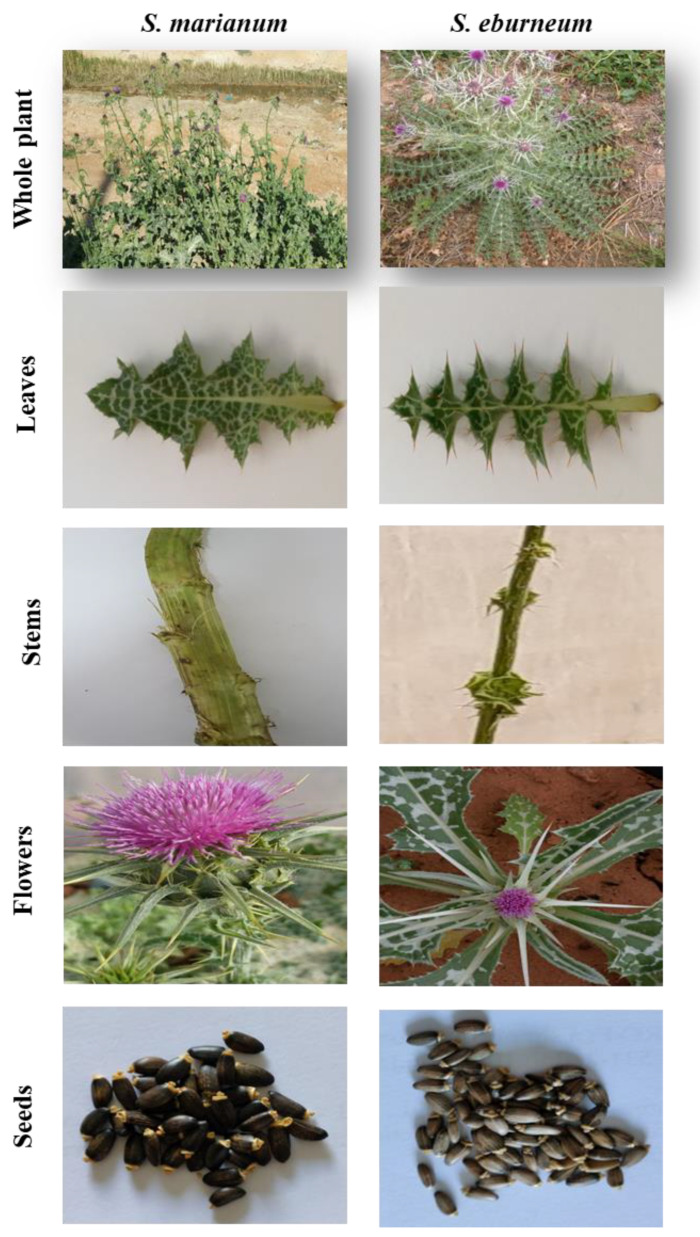
Different organs of *Silybum marianum* and *Silybum eburneum*.

The plant material used included the plants *Silybum marianum* and *Silybum eburneum* (leaves, stems, flowers, and mature and immature seeds). The species were identified botanically by Prof. Dr. Mohamed Tarhouni, a researcher at the Arid Regions Institute-Médenine, Tunisia. Voucher specimens were deposited at the herbarium of the Arid Regions Institute (IRA) with accession numbers IRAPL101 and IRAPL103 for *S. marianum* and *S. eburneum*, respectively. The *S. marianum* was harvested in Médenine (33°21′21.4″ N, 10°29′05.6″ E), located in the southeast of Tunisia, while *S. eburneum* was harvested in Sidi Bouzid (35°04′42″ N, 9°20′06″ E), located in the center of Tunisia. Twenty plants per species were randomly selected and collected in March 2021, while the collection of mature and immature seeds, gathered from twenty randomly selected plants per species, was carried out in May 2021. After harvesting, the plants were washed and separated into organs: leaves, stems, flowers, and seeds (Figure 2). The different plant parts of both species were finely ground into a powder for further use. For the seeds, the powder used was a defatted powder that was obtained after the extraction of fatty acids using the Soxhlet method.

### 4.2. Minerals Analysis

For the minerals analysis, 50 mL of 0.5% nitric acid was added to 50 mg of each sample. The mixtures were left to stand for 48 h in darkness with agitation. Afterward, the resulting extracts were filtered, and the concentrations of the mineral elements were determined using an atomic absorption spectrophotometer (Shimadzu AA-6800, Kyoto, Japan) that was equipped with WIZAARD 2.30 control software. The concentration of mineral components was calculated by referencing calibration curves specific to each element [53].

### 4.3. Soluble Sugar Content

The soluble sugar content was determined as detailed in ref. [54]. A 200 mg sample was ground in 1 mL of 80% ethanol, followed by incubation at 80 °C for 20 min. Afterward, it was centrifuged at 13,000× *g* for 20 min. This extraction process was repeated twice to maximize sugar extraction. The resulting mixed supernatant was stored at 4 °C until needed. Subsequently, the soluble sugar content was quantified using the high-performance liquid chromatography (HPLC) method (Shimadzu UFLC XR, Kyoto, Japan) with a refractive index detector (RID 10A) in isocratic mode. A total of 15 µL of each extract was injected into a NH_2_ amide column (4.6 μm, 4.6 × 250 mm) at an oven temperature of 45 °C. The pumps used were the “LC-20ADXR” type and the total flow rate of the mobile phase was 0.55 mL/min. The mobile phase used was water/ACN (17/83). Sigma-Aldrich sugar standards were used for HPLC. The soluble sugar content was expressed as milligrams per gram of dry weight (mg/g DW).

### 4.4. Organic Acid Content

The organic acid composition in the different parts of *Silybum* species was determined as previously detailed in ref. [54]. In brief, 200 mg per sample was ground in 1 mL of ultrapure distilled water and was then incubated at 80 °C for 1 h. After incubation, the samples were centrifugated at 11,000× *g* for 20 min, and the resulting supernatants were collected and stored at 4 °C until needed. The analysis of these extracts was carried out using the high-performance liquid chromatography (HPLC) method. The HPLC analysis was performed using an ultra-fast liquid chromatography system consisting of an LC-20AD XR binary pump system, SIL-20AC XR autosampler, CTO-20AC column oven, and DGU-20AS degasser, with the diode array detector SPD M20A (Shimadzu, Kyoto, Japan). An Agilent Hi-Plex H column with dimensions of 7.7 mm × 300 mm and 8 µm was used for analysis. The mobile phase consisted of 0.1 M H_2_SO_4_ in H_2_O. The flow rate of the mobile phase was 0.6 mL/min, the column temperature was maintained at 50 °C, and the injection volume was 5 µL. Chromatograms were monitored at 210 nm wavelengths and processed using Shimadzu LabSolutions software version 5.42. Chemical standards (oxalic acid, citric acid, malic acid, quinic acid, succinic acid, lactic acid, formic acid, acetic acid, propionic acid, and butyric acid) at a purity of 98% were purchased from Sigma Chemical Co. (St. Louis, MO, USA). The contents of organic acids were expressed as mg/g DW.

### 4.5. Storage Protein Content

The extraction of the four storage protein fractions from mature and immature seeds of *S. marianum* and *S. eburneum* was conducted using the method of ref. [55], slightly modified as detailed in refs. [56,57]. A total of 20 mg of defatted seed powder was homogenized in 2 mL distilled water (pH = 6.5) and centrifuged at 10,000 rpm for 10 min at 4 °C to extract the albumin fraction. The pellet was reextracted by adding 2 mL Tris-HCl buffer containing 100 mM Tris-HCl and 0.5 M NaCl (pH 8.1) to obtain the globulin fraction. Likewise, the prolamin fraction was extracted with a 55% (*v/v*) aqueous isopropanol solution and the glutelin fraction was extracted using a 0.2 M acetic acid solution. The content of the fractions was evaluated following the procedure outlined by ref. [58], using a standard range of Bovine Serum Albumin (B.S.A). The amounts of albumin, globulin, prolamin, and glutelin fractions were measured in mg/g DW.

### 4.6. Secondary Metabolite Screening

#### 4.6.1. Extraction of Active Ingredients

Weights of 10 g of powdered leaves, stems, flowers, and mature and immature seeds were extracted with 100 mL pure methanol by cold maceration for 24 h. The mixtures were centrifugated at 4500 rpm for 15 min and the obtained supernatants were filtered through a 0.2 µm syringe PTFE membrane filter. The solvents were evaporated under reduced pressure at 35 °C using a rotary vacuum evaporator. The resulting residues were stored in sterile glass bottles under refrigeration until they were utilized.

#### 4.6.2. Total Polyphenol and Flavonoid Contents

The total phenolic and flavonoid contents in the different plant organs were colorimetrically evaluated using the conventional Folin–Ciocalteu and aluminum chloride methods [59]. For the polyphenols, to a volume of 125 µL of plant extract, 500 µL of distilled water, 125 µL of Folin–Ciocalteu reagent, and 1250 mL Na_2_CO_3_ (7%) were added. To bring the total volume to 3 mL, distilled water was added, and the mixtures were then incubated for 90 min in darkness. After incubation, the absorbance of the samples was measured at a wavelength of 765 nm. The results obtained were expressed as milligrams of gallic acid equivalents per gram of dry weight (mg GAE/g DE). For flavonoids, 75 µL of NaNO_2_ (5%) and 150 µL of freshly prepared AlCl_3_ (10%) were mixed with 250 µL of plant extract. After a 5 min incubation, 500 µL of NaOH (4%) was added to the resulting mixture, and the final volume was adjusted to 3 mL with distilled water. The absorbance of the samples was measured at 510 nm. The total flavonoid content was expressed as milligrams of quercetin equivalents per gram of dry weight (mg QE/g DE).

#### 4.6.3. Characterization of Extracts by LC-ESI/MS

The analysis of phenolic compounds was conducted using a Shimadzu UFLC XR system (Kyoto, Japan) equipped with a SIL-20AXR autosampler, CTO-20 AC column oven, LC-20AD XR binary pump, and quadripole 2020 detector system. This instrument was fitted with an Inertsil ODS-4 C18 3 µm column. The column temperature was set at 40 °C and the injection volume was 20 µL with a flow rate of 0.5 mL/min. The mobile phases A and B were composed of 95% water + 5% MeOH + 0.2% acetic acid and 50% ACN + 50% water + 0.2% acetic acid, respectively. The analysis was carried out using a linear gradient programmed as follows: 0–14 min, from 10% to 20% B; 14–27 min, from 20% to 55% B; 27–37 min, from 55% to 100% B; 37–45 min, 100% B; 45–50 min, 10% B. The dissolving line temperature was 275 °C, the nebulizing gas flow was 1.50 L/min, and the drying gas was set at 15.00 L/min with a heat block temperature of 450 °C. LC-ESI(–)MS mass spectra [M–-H] were acquired using LabSolutions software. The identification of compounds was accomplished by comparing their retention time and mass spectra with those of reference standards [60]. The validation of the HPLC-ESI-MS method was achieved as detailed in ref. [61] in terms of sensitivity, linearity, and precision. The limits of detection (LODs) and quantification (LOQs) were determined using signal-to-noise ratios of 3 and 10 determinations, respectively, and after 3 injections of the lowest concentration. The linearity was evaluated by determining the correlation coefficients (R^2^) [62].

### 4.7. Antioxidant Potential

#### 4.7.1. Total Antioxidant Activity

The total antioxidant capacity (TAC) was investigated through the phosphomolybdenum assay, as detailed by ref. [28]. To 200 µL of sample extract, 2 mL of reagent solution composed of sulfuric acid (0.6 M), sodium phosphate (28 mM), and ammonium molybdate (4 mM) was added and the resulting mixture was incubated for 90 min at 95 °C. After cooling, the absorbance was determined at 700 nm versus a blank. The results were expressed as equivalents of gallic acid per gram of dry weight (mg GAE/g DE).

#### 4.7.2. DPPH Anti-Radical Activity

The antiradical potential effects of the different plant parts were evaluated against the DPPH free radical according to ref. [29]. A volume of 50 µL of each sample’s solution was mixed with 1.95 mL of 0.025 g/L DPPH solution. The obtained mixture was vigorously mixed and left to incubate for 30 min in the dark at room temperature. The reaction was monitored at 517 nm. The percentage of DPPH radical scavenging inhibition was measured as outlined below:

$$\frac{AC-AE}{AC}\times 100$$

where *AC* refers to the control absorbance value and *AE* refers to the plant extract absorbance value. The results were expressed as Trolox equivalent per gram of dry weight (mg TRE/g DE).

#### 4.7.3. Reducing Power Potential

The reducing power in the different plant parts was estimated using the FRAP method detailed in ref. [30]. In brief, 2.5 mL of phosphate buffer (0.2 M, pH = 6.6) and 2.5 mL of potassium ferricyanide (1%) were added to tubes that contained 1 mL of each extract. The tubes were incubated for 20 min at 50 °C, followed by the addition of 2.5 mL of 10% TCA. After centrifugation at 13,000 rpm for 10 min, 2.5 mL of the resulting supernatant was diluted with an equal volume of distilled water and mixed with 0.5 mL of FeCl_3_ solution (0.1), and the absorbance was measured at 700 nm. The reducing power in the extracts was evaluated as the Trolox equivalent per gram of dry weight (mg TRE/g DE).

### 4.8. Statistical Analysis

The statistical analysis was carried out using IBM SPSS statistical software (version 20.0, IBM Corp., Armonk, NY, USA). The data were presented as the mean ± standard deviation (SD) from three replicates and subjected to analysis of variance (ANOVA). To compare the means, the S-N-K post-hoc test was performed. Student’s *t* test was conducted to determine the significantly different effects of species and organs and their combined effects. The significance level was set at 5% to determine the differences between means.

## 5. Conclusions

In this study, the mineral, organic acid, free sugar, protein, and phytochemical contents as well as the antioxidant potentials of the stems, leaves, flowers, immature seeds, and mature seeds of wild edible *S. marianum* and *S. eburneum* were investigated. A significant difference in the effects of species, organs, and their interaction was shown in the major analyzed effects. The different organs showed higher contents of minerals including sodium, potassium, calcium, and magnesium. Additionally, *S. marianum* showed substantial fructose variation among its organs, with the highest contents in the stems. Conversely, *S. eburneum* exhibited significant heterogeneity in glucose, sucrose, and maltose levels across its organs. A notable organ-dependent distribution of organic acids was observed for oxalic, quinic, citric, malic, succinic, lactic, formic, acetic, and propionic acids among the two species. The colorimetric analysis revealed higher levels of polyphenol and flavonoid contents in both mature and immature seeds in both species compared to the other plant parts. A total of 32 polyphenolic compounds were identified through LC-ESI/MS, with 3,4-di-O-caffeoylquinic acid, 4,5-di-O-caffeoylquinic acid, syringic acid, protocatechuic acid, naringenin, apigenin, and quercetin detected as the dominant compounds. In both *S. marianum* and *S. eburneum*, albumins and globulins were the predominant protein fractions in both mature and immature seeds. In addition, the data obtained show important antioxidant properties of *S. marianum* and *S. eburneum* that could have major applications in nutritherapy, especially to prevent age-related diseases often associated with a rupture of the RedOx status [63]. This study also highlights the value of *Silybum* as a sustainable plant source with significant potential for nutritional applications in the production of functional foods in various sectors of the food industry.

## Figures and Tables

**Table 1:** Mineral composition (mg/100 g DW) of *Silybum marianum* (SM) and *Silybum eburneum* (SE) plant parts.

		Na	K	Ca	Mg	Cu	Fe	Mn
SM	L	18.7 ± 2.1 ^A^	39.6 ± 6.3 ^B^	154.5 ± 6.1 ^B^	5.1 ± 0.3 ^A^	0.04 ± 0.01 ^B^	0.4 ± 0.03 ^A^	0.07 ± 0.002 ^A^
	S	19.8 ± 3.3 ^A^	44.2 ± 8.3 ^A^	100.5 ± 8.3 ^C^	3.3 ± 0.2 ^C^	0.1 ± 0.01 ^A^	0.2 ± 0.03 ^C^	0.05 ± 0.003 ^B^
	Fl	9.9 ± 0.9 ^B^	27.2 ± 0.9 ^C^	166.3 ± 1.0 ^B^	3.9 ± 0.3 ^BC^	0.1 ± 0.01 ^A^	0.3 ± 0.02 ^B^	0.05 ± 0.003 ^B^
	M.S	6.9 ± 6.8 ^B^	8.1 ± 1.0 ^D^	114.0 ± 31.7 ^C^	5.1 ± 0.8 ^A^	0.1 ± 0.02 ^A^	0.2 ± 0.03 ^B^	0.05 ± 0.01 ^B^
	I.S	6.8 ± 0.4 ^B^	11.4 ± 0.4 ^D^	326.9 ± 9.1 ^A^	4.5 ± 0.3 ^AB^	0.1 ± 0.01 ^A^	0.2 ± 0.02 ^B^	0.06 ± 0.01 ^B^
SE	L	50.9 ± 3.3 ^a^	56.3 ± 11.1 ^b^	224.6 ± 30.0 ^a^	24.2 ± 5.4 ^a^	0.05 ± 0.01 ^ab^	0.3 ± 0.1 ^a^	0.1 ± 0.004 ^a^
	S	58.3 ± 17.8 ^a^	103.4 ± 22.5 ^a^	178.9 ± 18.3 ^a^	21.0 ± 2.6 ^ab^	0.02 ± 0.001 ^b^	0.2 ± 0.02 ^a^	0.1 ± 0.01 ^a^
	Fl	64.9 ± 3.4 ^a^	93.6 ± 5.7 ^a^	230.2 ± 21.7 ^a^	13.5 ± 1.8 ^c^	0.1 ± 0.02 ^a^	0.3 ± 0.1 ^a^	0.1 ± 0.01 ^a^
	M.S	41.4 ± 8.8 ^a^	54.7 ± 8.1 ^b^	106.9 ± 28.3 ^b^	16.1 ± 0.7 ^bc^	0.1 ± 0.03 ^a^	0.3 ± 0.1 ^a^	0.1 ± 0.02 ^a^
	I.S	48.2 ± 13.2 ^a^	46.2 ± 8.8 ^b^	116.3 ± 47.2 ^b^	16.7 ± 1.9 ^bc^	0.1 ± 0.03 ^a^	0.3 ± 0.01 ^a^	0.04 ± 0.02 ^a^

Values are expressed as mean ± SD (n = 3). The same upper letters in the same column show no significant difference between organs of *S. marianum* plant and the same lower letters in the same column show no significant difference between organs of *S. eburneum* plant (*p* < 0.05; S.N.K test). SM: Silybum marianum; SE: Silybum eburneum; L: leaf; S: stem; Fl: flower; M.S: mature seed; I.S: immature seed.

**Table 2:** Content of free sugars (mg/g DW) in the organs of *Silybum marianum* (SM) and *Silybum eburneum* (SE).

		Fructose	Glucose	Sucrose	Maltose
SM	L	0.3 ± 0.02 ^C^	12.1 ± 3.5 ^A^	0.1 ± 0.02 ^B^	ND
	S	3.5 ± 0.4 ^A^	17.7 ± 14.8 ^A^	0.2 ± 0.01 ^B^	ND
	Fl	1.1 ± 0.1 ^B^	4.2 ± 0.3 ^A^	0.2 ± 0.16 ^B^	ND
	M.S	0.01 ± 0.01 ^C^	0.5 ± 0.1 ^B^	0.6 ± 0.02 ^A^	ND
	I.S	0.02 ± 0.01 ^C^	ND	0.1 ± 0.02 ^B^	ND
SE	L	5.0 ± 1.3 ^a^	3.8 ± 1.0 ^a^	3.3 ± 2.8 ^a^	ND
	S	5.5 ± 1.3 ^a^	0.8 ± 0.7 ^b^	1.6 ± 0.4 ^a^	ND
	Fl	4.8 ± 0.3 ^a^	1.6 ± 0.3 ^b^	1.1 ± 0.7 ^a^	ND
	M.S	0.3 ± 0.1 ^b^	3.6 ± 0.8 ^a^	2.7 ± 0.3 ^a^	ND
	I.S	0.6 ± 0.01 ^b^	0.2 ± 0.03 ^b^	3.4 ± 0.4 ^a^	2.3 ± 0.2

Values are expressed as mean ± SD (n = 3). The same upper letters in the same column show no significant difference between organs of *S. marianum* plant and the same lower letters in the same column show no significant difference between organs of *S. eburneum* plant (*p* < 0.05; S.N.K test). SM: *Silybum marianum;* SE: *Silybum eburneum*; L: leaf; S: stem; Fl: flower; M.S: mature seed; I.S: immature seed; ND: not detected.

**Table 3:** *p*-values of the statistical analyses (Student’s *t* test) related to the effects of species, organs, and species–organs interaction on the phytochemical profiles and antioxidant activities of *S. marianum* and *S. eburneum*.

	*p*-Values		
	Species	Organs	Species × Organs
*Antioxidant*			
Total phenol content	<0.0001	<0.0001	<0.0001
Total flavonoid content	<0.0001	<0.0001	<0.0001
Total antioxidant activities	<0.0001	<0.0001	<0.0001
DPPH	0.001	<0.0001	<0.0001
FRAP	<0.0001	<0.0001	<0.0001
*Sugars*			
Fructose	<0.0001	<0.0001	<0.0001
Glucose	0.034	0.059	0.059
Sucrose	<0.0001	0.176	0.132
Maltose	<0.0001	<0.0001	<0.0001
*Organic acids*			
Oxalic acid	<0.0001	0.001	0.001
Quinic acid	<0.0001	<0.0001	<0.0001
Citric acid	<0.0001	<0.0001	<0.0001
Malic acid	<0.0001	<0.0001	<0.0001
Succinic acid	<0.0001	<0.0001	<0.0001
Lactic acid	<0.0001	<0.0001	<0.0001
Formica cid	0.005	<0.0001	<0.0001
Acetic acid	0.075	<0.0001	0.039
Propionic acid	0.005	<0.0001	<0.0001
*Minerals*			
Na	<0.0001	0.015	0.141
K	<0.0001	<0.0001	0.002
Ca	0.912	<0.0001	<0.0001
Mg	<0.0001	0.002	0.002
Cu			
Fe			
Mn			
*Polyphenolic compounds*			
Gallic acid	0.323	0.416	0.486
Protocatechuic acid	0.055	0.675	0.606
30.4-di-O-caffeoyquinic acid	<0.0001	<0.0001	<0.0001
40.5-di-O-caffeoyquinic acid	<0.0001	<0.0001	<0.0001
Quinic acid	<0.0001	<0.0001	<0.0001
10.3-di-O-caffeoyquinic acid	0.029	<0.0001	0.050
Salviolinic acid	<0.0001	<0.0001	<0.0001
Chlorogenic acid	0.059	0.015	0.015
Trans ferulic acid	<0.0001	<0.0001	<0.0001
Syringic acid	0.151	0.604	0.611
p-coumaric acid	0.106	0.656	0.688
Rosmarinic acid	<0.0001	<0.0001	<0.0001
o-coumaric acid	0.162	0.587	0.623
Caffeic acid	<0.0001	<0.0001	<0.0001
Salviolinic acid	<0.0001	<0.0001	<0.0001
Trans cinnamic acid	0.532	0.118	0.161
Luteolin	0.185	0.551	0.570
Cirsilineol	0.173	0.570	0.569
Rutin	0.284	0.453	0.453
Luteolin-7-o-glucoside	0.305	0.446	0.444
Epicatechin	0.453	0.353	0.479
Acacetin	0.299	0.450	0.450
Catechin (+)	0.004	<0.0001	<0.0001
Cirsiliol	0.191	0.490	0.490
Quercetin-3-o-galactoside	0.979	0.315	0.101
Naringin	0.046	0.092	0.092
Quercetrin	0.021	0.002	0.002
Apegenin-7-o-glucoside	0.329	0.431	0.431
Kaempferol	0.329	0.431	0.431
Quercetin	<0.0001	<0.0001	<0.0001
Naringenin	<0.0001	<0.0001	<0.0001
Apigenin	<0.0001	<0.0001	<0.0001

**Table 4:** Organic acid contents (mg/g DW) of *Silybum marianum* (SM) and *Silybum eburneum* (SE) organs.

		Oxalic Acid	Quinic Acid	Citric Acid	Malic Acid	Succinic Acid	Lactic Acid	Formic Acid	Acetic Acid	Propionic Acid
SM	L	ND	ND	6.5 ± 1.3	15.0 ± 1.6 ^A^	1.0 ± 0.1 ^A^	0.6 ± 0.03 ^B^	0.2 ± 0.04 ^C^	37.3 ± 2.3 ^A^	0.5 ± 0.03 ^A^
	S	ND	ND	ND	5.2 ± 0.6 ^C^	0.1 ± 0.04 ^B^	0.2 ± 0.1 ^B^	0.04 ± 0.01 ^D^	1.9 ± 0.7 ^B^	0.2 ± 0.002 ^C^
	Fl	ND	ND	ND	11.1 ± 3.3 ^B^	0.2 ± 0.1 ^B^	0.6 ± 0.4 ^B^	0.2 ± 0.01 ^C^	2.1 ± 0.4 ^B^	0.4 ± 0.1 ^B^
	M.S	ND	ND	ND	0.3 ± 0.02 ^D^	ND	0.3 ± 0.01 ^B^	0.5 ± 0.01 ^A^	0.4 ± 0.01 ^B^	0.2 ± 0.02 ^C^
	I.S	ND	ND	ND	1.7 ± 0.1 ^D^	ND	3.7 ± 0.1 ^A^	0.3 ± 0.05 ^B^	0.5 ± 0.04 ^B^	0.2 ± 0.02 ^C^
SE	L	0.03 ± 0.02 ^a^	0.7 ± 0.3 ^b^	0.2 ± 0.1 ^ab^	0.45 ± 0.1 ^ab^	1.2 ± 0.2 ^a^	ND	0.2 ± 0.03 ^c^	38.8 ± 3.8 ^a^	0.4 ± 0.1 ^b^
	S	0.003 ± 0.002 ^b^	1.1 ± 0.2 ^a^	0.3 ± 0.1 ^a^	0.3 ± 0.1 ^bc^	ND	ND	0.2 ± 0.01 ^c^	0.2 ± 0.04 ^c^	ND
	Fl	ND	1.1 ± 0.1 ^a^	0.2 ± 0.04 ^ab^	0.6 ± 0.2 ^a^	0.9 ± 0.3 ^b^	ND	0.2 ± 0.03 ^c^	6.4 ± 1.5 ^b^	0.5 ± 0.1 ^b^
	M.S	0.01 ± 0.001 ^ab^	0.2 ± 0.02 ^c^	0.1 ± 0.03 ^b^	0.1 ± 0.01 ^c^	0.5 ± 0.1 ^c^	0.2 ± 0.02 ^a^	0.3 ± 0.1 ^b^	0.6 ± 0.04 ^c^	ND
	I.S	0.02 ± 0.01 ^a^	ND	0.1 ± 0.01 ^b^	0.2 ± 0.03 ^c^	ND	0.2 ± 0.08 ^a^	0.4 ± 0.03 ^a^	1.4 ± 0.3 ^c^	1.0 ± 0.2 ^a^

Values are expressed as mean ± SD (n = 3). The same upper letters in the same column show no significant difference between organs of *S. marianum* plant and the same lower letters in the same column show no significant difference between organs of *S. eburneum* plant (*p* < 0.05; S.N.K test). ND: not detected; SM: *Silybum marianum*; SE: *Silybum eburneum*; L: leaf; S: stem; Fl: flower; M.S: mature seed; I.S: immature seed.

**Table 5:** Total phenol and flavonoid contents and antioxidant activities of *Silybum marianum* (SM) and *Silybum eburneum* (SE) organs.

		Total Phenol Content (mg GAE/g DE)	Flavonoid Content (mg QRE/g DE)	Antioxidant Activity		
				TAA (mg GAE/g DE)	DPPH (mg TRE/g DE)	FRAP (mg TRE/g DE)
SM	L	1.7 ± 0.1 ^D^	7.4 ± 1.0 ^D^	19.9 ± 2.0 ^C^	0.8 ± 0.1 ^D^	1.4 ± 0.1 ^D^
	S	0.7 ± 0.1 ^E^	2.9 ± 0.3 ^E^	8.1 ± 0.7 ^D^	0.5 ± 0.03 ^E^	1.3 ± 0.2 ^D^
	Fl	5.9 ± 0.7 ^C^	12.3 ± 0.5 ^C^	28.6 ± 2.4 ^A^	1.5 ± 0.2 ^C^	3.9 ± 0.2 ^C^
	M.S	161.4 ± 13.9 ^A^	41.9 ± 0.4 ^A^	24.7 ± 0.4 ^B^	3.4 ± 0.004 ^B^	44.9 ± 1.1 ^A^
	I.S	55.9 ± 8.3 ^B^	23.9 ± 1.1 ^B^	31.2 ± 0.5 ^A^	4.4 ± 0.01 ^A^	31.4 ± 3.1 ^B^
SE	L	1.4 ± 0.2 ^e^	3.6 ± 0.2 ^d^	5.8 ± 0.3 ^b^	0.6 ± 0.1 ^c^	1.6 ± 0.3 ^e^
	S	4.7 ± 1.6 ^d^	7.9 ± 2.5 ^c^	3.9 ± 2.0 ^b^	1.5 ± 0.4 ^b^	3.3 ± 0.6 ^d^
	Fl	24.6 ± 6.0 ^c^	16.9 ± 2.0 ^a^	22.6 ± 1.1 ^a^	2.5 ± 0.3 ^a^	8.1 ± 0.9 ^c^
	M.S	57.9 ± 4.6 ^a^	17.5 ± 1.5 ^a^	5.9 ± 0.4 ^b^	2.1 ± 0.01 ^a^	16.0 ± 0.5 ^a^
	I.S	41.7 ± 8.0 ^b^	13.2 ± 0.3 ^b^	4.6 ± 0.4 ^b^	2.5 ± 0.1 ^a^	9.7 ± 0.6 ^b^

Values are expressed as mean ± SD (n = 3). The same upper letters in the same column show no significant difference between organs of *S. marianum* plant and the same lower letters in the same column show no significant difference between organs of *S. eburneum* plant (*p* < 0.05; S.N.K test). TAA: total antioxidant activity; GAE: gallic acid equivalents; QRE: quercetin equivalents; TRE: Trolox equivalence; DE: dry extract; SM: *Silybum marianum*; SE: *Silybum eburneum*; L: leaf; S: stem; Fl: flower; M.S: mature seed; I.S: immature seed.

**Table 6:** Phenolic acid profiles (µg/g DE) of *Silybum marianum* and *Silybum eburneum* organs identified by LC-ESI/MS analysis.

	*Sylibum marianum* (SM)					*Sylibum eburneum* (SE)				
	L	S	Fl	M.S.	I.S.	L	S	Fl	M.S.	I.S.
-Gallic acid	ND	ND	ND	ND	ND	ND	ND	2.9 ± 0.7 ^b^	0.7 ± 0.2 ^c^	3.3 ± 0.7 ^a^
-Protocatechuic acid	326.4 ± 65.3 ^B^	ND	4133.5 ± 826.7 ^A^	ND	ND	ND	20.3 ± 15.9 ^b^	56.7 ± 16.6 ^a^	4.6 ± 0.2 ^c^	3.6 ± 0.7 ^d^
-3,4-di-O-caffeoylquinic acid	11405.2 ± 135.1 ^B^	3402.4 ± 10.5 ^C^	20538 ± 3109 ^A^	ND	ND	ND	ND	ND	ND	ND
-4,5-di-O-caffeoylquinic acid	2269.1 ± 58 ^A^	503.2 ± 12.58 ^B^	ND	ND	ND	ND	ND	ND	ND	ND
-Quinic acid	72.1 ± 5.1 ^E^	104.0 ± 3.9 ^D^	357.9 ± 49.9 ^C^	10404.5 ± 390.2 ^A^	6389.3 ± 127.9 ^B^	4.6 ± 0.8 ^e^	16.01 ± 5.9 ^d^	34.2 ± 4.9 ^c^	64.6 ± 15 ^b^	107.5 ± 24.5 ^a^
-1,3-di-O-caffeoylquinic acid	3.5 ± 0.6 ^A^	2.2 ± 1.8 ^B^	ND	ND	ND	2.6 ± 0.2 ^b^	ND	7.4 ± 1.3 ^a^	ND	ND
-Salviolinic acid	19.2 ± 0.4	ND	ND	ND	ND	ND	ND	ND	ND	ND
-Chlorogenic acid	ND	ND	ND	ND	ND	82.4 ± 18.8 ^a^	ND	33.9 ± 6.4 ^b^	ND	ND
-Trans ferulic acid	5.9 ± 0.6 ^C^	ND	58.3 ± 7 ^B^	1103.9 ± 132.5 ^A^	ND	0.2 ± 0.01 ^b^	0.7 ± 0.3 ^a^	0.8 ± 0.1 ^a^	ND	ND
-Syringic acid	103.6 ± 22.8 ^B^	ND	1374.3 ± 189 ^A^	ND	ND	13.2 ± 4.7 ^c^	168.1 ± 28.9 ^a^	110.02 ± 10.7 ^b^	ND	6.02 ± 1.3 ^d^
-P-coumaric acid	207.8 ± 10.4 ^B^	ND	ND	ND	2663.9 ± 133.2 ^A^	0.5 ± 0.04 ^d^	0.2 ± 0.1 ^e^	3.2 ± 0.3 ^a^	2.1 ± 0.04 ^b^	1.7 ± 0.1 ^c^
-Rosmarinic acid	ND	114.2 ± 61.6	ND	ND	ND	ND	ND	ND	ND	ND
-O-coumaric acid	ND	214.4 ± 134.6 ^B^	29.4 ± 5.9 ^C^	1175.2 ± 176.3 ^A^	ND	ND	ND	ND	ND	ND
-Caffeic acid	ND	27.6	ND	ND	ND	ND	ND	ND	8.2 ± 0.6 ^a^	0.8 ± 0.4 ^b^
-Salviolinic acid	ND	Nd	6.2 ± 0.5	ND	ND	ND	ND	ND	ND	ND
-Trans cinnamic acid	482.1 ± 43.4 ^B^	Nd	ND	7319.9 ± 658.8 ^A^	ND	ND	ND	ND	2.1 ± 0.3 ^b^	7.5 ± 0.8 ^a^
-Luteolin	3.8 ± 0.6 ^D^	Nd	24.8 ± 2.7 ^C^	118.3 ± 20.1 ^A^	126.8 ± 21.6 ^A^	2.1 ± 0.2 ^b^	ND	68.7 ± 12 ^a^	ND	ND
-Cirsilineol	128 ± 19.2 ^B^	181.4 ± 1.8 ^A^	ND	ND	ND	4.7 ± 0.7	ND	ND	ND	ND
-Rutin	5.4 ± 0.3 ^C^	1.7 ± 0.1 ^D^	14.6 ± 2.2 ^B^	ND	509.1 ± 76.4 ^A^	ND	ND	ND	ND	ND
-Luteolin-7-o-glucoside	4.9 ± 0.8 ^A^	4.7 ± 0.7 ^A^	ND	ND	ND	16.9 ± 7.1 ^b^	20.9 ± 5.9 ^a^	ND	1.2 ± 0.2 ^c^	ND
-Epicatechin	ND	157.0 ± 31.4 ^B^	216.4 ± 58 ^A^	ND	ND	ND	ND	ND	ND	ND
-Acacetin	ND	ND	32.8 ± 22.7	ND	ND	ND	ND	ND	ND	ND
-Catechin (+)	ND	ND	6.8 ± 0.5	ND	ND	ND	ND	ND	ND	ND
-Cirsiliol	ND	ND	ND	ND	ND	0.4 ± 0.1 ^d^	18.2 ± 10.1 ^b^	ND	10.8 ± 0.5 ^c^	77.8 ± 3.7 ^a^
-Quercetin-3-o-galactoside	18.4 ± 2.1 ^D^	10.4 ± 6.3 ^E^	101.9 ± 11.9 ^C^	194.1 ± 27.2 ^B^	454.0 ± 77.2 ^A^	6.1 ± 0.9 ^b^	4.4 ± 1.9 ^c^	ND	ND	454.0 ± 90.8 ^a^
-Naringin	28.9 ± 4.8 ^B^	4.8 ± 2.3 ^C^	50.7 ± 11.1 ^A^	ND	ND	ND	34.1 ± 10.8	ND	ND	ND
-Quercetrin	16.9 ± 2.7 ^C^	3.2 ± 0.4 ^D^	266.2 ± 20.6 ^A^	ND	226.8 ^B^	10.4 ± 0.7 ^c^	21.9 ± 5.3 ^b^	140.7 ± 40.4 ^a^	ND	ND
-Apegenin-7-o-glucoside	3.8 ± 1.6 ^C^	1.3 ± 0.02 ^D^	ND	8.9 ± 0.5 ^B^	30.5 ± 2.1 ^A^	63.6 ± 0.9 ^b^	139.9 ± 54.1 ^a^	ND	3.3 ± 0.2 ^c^	2.5 ± 0.1 ^d^
-Kaempferol	5.01 ± 1.2 ^D^	ND	19.8 ± 14.5 ^C^	152.6 ± 30.5 ^B^	198.1 ± 27.7 ^A^	17.3 ± 0.5 ^b^	ND	1281.3 ± 82.2 ^a^	ND	ND
-Quercetin	3.4 ± 0.2 ^E^	104.7 ± 139.6 ^C^	5.3 ± 3.1 ^D^	1470.9 ± 250.0 ^B^	1814.9 ± 326.7 ^A^	ND	1.4 ± 0.04 ^b^	ND	ND	3.9 ± 0.1 ^a^
-Naringenin	141.2 ± 19.7 ^D^	24.0 ± 2.2 ^E^	469.9 ± 140.5 ^C^	3091.5 ± 401.9 ^A^	1694.4 ± 203.2 ^B^	11.7 ± 1.2 ^c^	9.8 ± 4.5 ^e^	160.1 ± 17.3 ^a^	26.5 ± 3.6 ^b^	10.1 ± 2.9 ^d^
-Apigenin	322.1 ± 124.7 ^C^	114.2 ± 73.4 ^D^	7077.5 ± 16.1 ^A^	ND	936.5 ± 84.3 ^B^	11.1 ± 1 ^c^	27.3 ± 7.1 ^b^	877.5 ± 158.6 ^a^	0.4 ± 0.1 ^e^	1.4 ± 0.05 ^d^
*-Detected compounds*	*22*	*18*	*19*	*10*	*11*	*16*	*14*	*13*	*11*	*13*

Values are expressed as mean ± SD. The same upper letters in the same column show no significant difference between organs of *S. marianum* plant and the same lower letters in the same column show no significant difference between organs of *S. eburneum* plant (*p* < 0.05; S.N.K test). SM: *Silybum marianum*; SE: *Silybum eburneum*; L: leaf; S: stem; Fl: flower; M.S: mature seed; I.S: immature seed; ND: not detected; DE: dry extract.

## Data Availability

The datasets used and/or analyzed in the current study are available from the corresponding author upon reasonable request.
